# Pericardial Effusion as a Presenting Symptom of Hashimoto Thyroiditis: A Case Report

**DOI:** 10.3390/ijerph14121576

**Published:** 2017-12-14

**Authors:** Alberto Leonardi, Laura Penta, Marta Cofini, Lucia Lanciotti, Nicola Principi, Susanna Esposito

**Affiliations:** 1 Paediatric Clinic, Department of Surgical and Biomedical Sciences, Università degli Studi di Perugia, 06132 Perugia, Italy; alberto.leonardi88@gmail.com (A.L.); laura.penta@ospedale.perugia.it (L.P.); marta.cofini@gmail.com (M.C.); lucia.lanciotti@gmail.com (L.L.); 2 Università degli Studi di Milano, 20122 Milan, Italy; nicola.principi@unimi.it

**Keywords:** Hashimoto thyroiditis, hypothyroidism, pleural effusion, thyroid

## Abstract

**Background:** Hashimoto thyroiditis (HT) is the most frequent cause of acquired hypothyroidism in paediatrics. HT is usually diagnosed in older children and adolescents, mainly in females and is rare in infants and toddlers with cardiac involvement, including pericardial effusion, that can be found in 10% to 30% of adult HT cases. In this paper, a child with HT and pericardial effusion as the most important sign of HT is described. **Case presentation**: A four-year-old male child suffering for a few months from recurrent abdominal pain sometimes associated with vomiting underwent an abdominal ultrasound scan outside the hospital. This led to the identification of a significant pericardial effusion. At admission, his family history revealed that both his mother and maternal grandmother suffered from HT and that both were treated with l-thyroxine (LT4). The clinical examination did not reveal any pathological signs other than a palpable thyroid. His weight was 21 kg (78th percentile), his height was 101.8 cm (12th percentile) and his body max index (BMI) was 20.26 (96th percentile). On a chest radiograph, his heart had a globular appearance and the lung fields were normal. An echocardiography confirmed and determined the effusion amount (max, 23 mm; 600 mL) with light impairment of the heart kinetics. The ECG showed sinus bradycardia with a normal ST tract. Based on the blood test results, an infectious cause of the pericardial fluid excess was considered unlikely. Thyroid function testing revealed very high thyrotropin (TSH, 487 μIU/mL; normal range, 0.340–5.600 μIU/mL) and low serum-free thyroxine (fT4, 0.04 ng/dL; normal range, 0.54–1.24 ng/dL) levels. High thyroid peroxidase antibody titres in the blood were evidenced (>1500 UI/L; normal values, 0.0–9.0 UI/L). The thyroid ultrasound was consistent with thyroiditis. HT was diagnosed, and LT4 replacement therapy with levothyroxine sodium 1.78 µg/kg/die was initiated, with a gradual increase of the administered dose. The treatment was successful because a complete regression of the effusion after one month was evidenced, with a substantial modification towards normality of the thyroid function tests. One year later, the substitutive therapy led to complete normalization of the thyroid function indexes. A slight reduction of weight (BMI, 17.60 for age) and an increase of the velocity of height growth were evidenced. **Conclusions**: When fluid is identified in the pericardial space and pericarditis of unknown origin is diagnosed, the thyroid function should be immediately evaluated to prescribe substitutive hormonal therapy if necessary and thereby avoid overt hypothyroidism development and the risk of cardiac tamponade.

## 1. Background

Hashimoto thyroiditis (HT) is the most frequent cause of acquired hypothyroidism in paediatrics, although in some cases it can be associated, particularly at presentation, with normal thyroid function or even with transient hyperthyroidism [1]. It is an autoimmune disease that can occur alone or be associated with other autoimmune disorders, mainly type 1 diabetes mellitus, causing the so-called autoimmune polyglandular syndromes [2]. HT is usually diagnosed in older children and adolescents, mainly in females, and is rare in infants and toddlers. The global incidence in paediatrics varies from 0.3% [3] to 9.6% [4], according to the methods used for diagnosis and the characteristics of the studied population. Genetic susceptibility and environmental factors favour disease development and can explain these differences. Moreover, an epigenetic mechanism has been suggested in some cases [5].

Systemic manifestations of HT can significantly vary according to the thyroid function, the presence of associated diseases and the duration of autoimmunity. Long-term overt hypothyroidism significantly affects growth as well as heart, liver, skin, and kidney functions, causing signs and symptoms that promptly lead to its diagnosis [1]. However, in some cases, children with HT have subclinical hypothyroidism or signs that are difficult to evaluate. This situation can lead to misdiagnosis and mismanagement. Cardiac involvement, including pericardial effusions, which can be found in 10% to 30% of adult HT cases [6], is not common in children. Frequently, HT is asymptomatic and is occasionally diagnosed [7,8]. In these patients, particularly when pericardial effusion is the only relevant sign of disease, the HT diagnosis remains very difficult. The effusion can increase uncontrolled for months and lead to more severe complications such as cardiac tamponade [9,10]. This means that attention should be paid to pericardial effusion with a poorly defined origin; moreover, in the presence of this clinical manifestation, HT should be excluded, and adequate hormonal therapy should be prescribed to avoid risks of a negative outcome. In this paper, a child with HT and pericardial effusion as the most important sign of HT is described.

## 2. Ethics, Approval and Consent to Participate

This case report was approved by the Ethics Committee of Azienda Ospedaliera di Perugia, Perugia, Italy. Azienda Ospedaliera di Perugia, Perugia, does not provide a reference number for case reports. Written informed consent for publishing this case report and any accompanying images were obtained from the patient’s parents. A copy of the written consent is available for review by the Editor-in-Chief of this journal.

## 3. Case Report

A four-year-old male child suffering for a few months from recurrent abdominal pain sometimes associated with vomiting underwent an abdominal ultrasound scan outside the hospital. This led to the identification of a significant pericardial effusion. To clarify the cause of this unexpected clinical problem, the child was hospitalized. At admission, his family history revealed that both the mother and the maternal grandmother suffered from HT and that both were treated with l-thyroxine (LT4). The clinical history of the child was negative as far as foetal life, birth, height, weight and neurologic and psychiatric development. No congenital or acquired disease, constipation, hair loss, or recent febrile illness were reported except for the abovementioned abdominal pain. For this symptom, routine blood tests, radioallergosorbent tests for common foods and celiac disease screening tests had already been performed and had yielded normal results.

**Figure 1:**
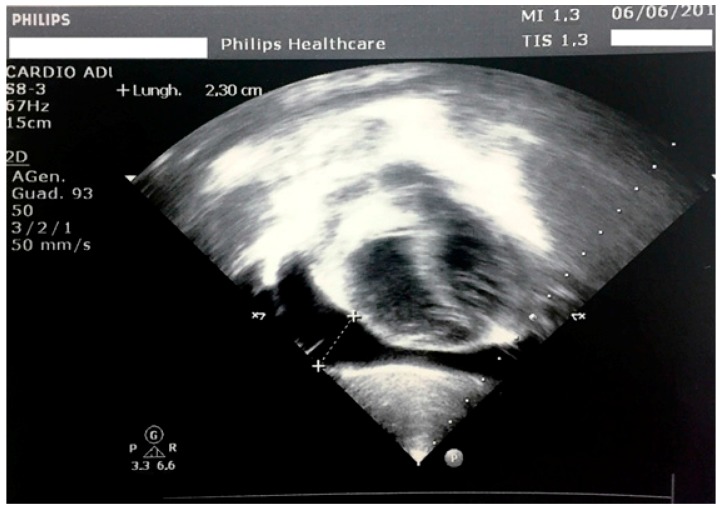
Echocardiography at admission, with evidence of a hyperechogenic pericardium and a large pericardial effusion.

In the hospital, the clinical examination did not reveal any pathological signs other than a palpable thyroid. He had a normal heart rhythm with an innocent systolic heart murmur without pericardial rubs. The lungs were clear to auscultation and percussion bilaterally. The abdomen was soft, nontender, and nondistended. No pathologic signs were identified in the neurologic examination. Vital signs showed a pulse of 80 bpm (<2 standard deviation (SD) for age), a respiratory rate of 16 breaths/minute, SpO2 of 100% in room air, temperature of 36.5 °C, and blood pressure of 94/57 mmHg. His weight was 21 kg (78th percentile, 0.78 SD), his height was 101.8 cm (12th percentile, −1.18 SD), and his body mass index (BMI) was 20.26 (96th percentile, 1.76 SD) according to the Italian Society for Paediatric Endocrinology and Diabetes charts [11]. The mid-parental height was 166.0 cm (−1.63 SD). His bone age was delayed by one year with respect to the chronological age. On the chest radiograph, the heart had a globular appearance, and the lung fields were normal. An echocardiography confirmed and determined the effusion amount (max, 23 mm; 600 mL) with light impairment of the heart kinetics (Figure 1).

**Figure 2:**
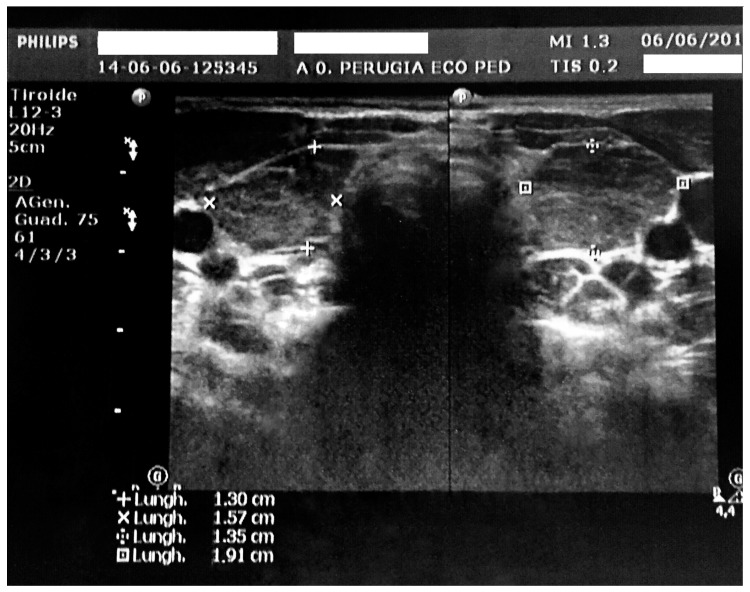
Thyroid ultrasound with evidence of increased gland dimensions and a non-homogeneous echotexture aspect.

The ECG showed sinus bradycardia with a normal ST tract. However, QT dispersion corrected for heart rate was longer than in normal subjects (90 ms). According to the European Society of Cardiology guidelines, it was possible to exclude acute pericarditis due to the absence of at least two major criteria among pericarditic chest pain, pericardial rubs, widespread ST-elevation or PR depression on ECG, and pericardial effusion [12]. As the duration of effusion was unknown, whether it was incessant, recurrent or chronic could not be evaluated. Pericardialcentesis was not performed because the child was in good clinical condition and could be continuously monitored. Based on the blood test results, an infectious cause of the pericardial fluid excess was considered unlikely. The white blood cell count was 5920/mm^3^, the lymphocyte count was 60.5%, the neutrophil count was 33.8%, and C-reactive protein (CRP) and the erythrocyte sedimentation rate were in the normal range. In addition, pharyngeal swabs for respiratory pathogens, the Mantoux test, and IgM antibodies against cytomegalovirus, Epstein-Barr virus, adenovirus, parvovirus B19, and *Mycoplasma pneumoniae* were all negative. General biochemical examinations revealed minor creatine phosphokinase (510 U/L) and aspartate aminotransferase (55 U/L) elevation. Serum electrolytes were in the normal range (Na 138 mEq/L, K 4.9 mEq/L, Cl 96 mEq/L) Renal function indexes and anti-nuclear antibodies (ANA) were found to be normal. Thyroid function testing revealed very high thyrotropin (TSH, 487 μIU/mL; normal range, 0.340–5.600 μIU/mL) and low serum-free thyroxine (fT4, 0.04 ng/dL; normal range, 0.54–1.24 ng/dL) levels. High thyroid peroxidase antibody titres in the blood were evidenced (>1500 UI/L; normal values, 0.0–9.0 UI/L). The thyroid ultrasound was consistent with thyroiditis. It evidenced a non-homogeneous echotexture with high vascularization and increased gland volume for age (almost five times the normal value, Figure 2).

HT was diagnosed, and LT4 replacement therapy with levothyroxine sodium 1.78 µg/kg/die was initiated, with a gradual increase of the administered dose. The treatment was successful because complete regression of the effusion after one month was evidenced, with a substantial modification towards normality of the thyroid function tests.

One year later, the substitutive therapy led to complete normalization of the thyroid function indexes (TSH, 2.96 µUI/mL; fT4, 1.25 ng/dL). The QT dispersion was also normalized, corrected for heart rate (71 ms). A slight reduction of weight (22 kg, 62th percentile, 0.31 SD) and BMI (17.60, 80th percentile, 0.83 SD) for age and an increase of height velocity growth (height, 111.8 cm; 33th percentile, −0.44 SD; velocity 9.99 cm/year, >97th percentile, 3.41 SD) were evidenced.

## 4. Discussion

HT is uncommon in preschool children. Moreover, in some cases, the disease presentation is unusual, leading to diagnostic pitfalls. Generally, in these patients, the final diagnosis is delayed and made only when frank clinical signs and symptoms of thyroid impairment develop. Pericardial effusion can be, although rarely, one of the presenting manifestations of paediatric HT with hypothyroidism. Reduced thyroid functioncauses increased protein extravasation and relatively slow lymphatic drainage. This leads to myxoedema with fluid accumulation in serous cavities, including the pericardial cavity [13]. Generally, this fluid accumulation is very slow, favouring heart adaptation to the gradual filling. This explains why, in some cases, this condition remains asymptomatic for a long time and becomes clinically evident mainly during the most advanced stage of HT when the myxoedema is significant and the clinical picture of overt hypothyroidism is fully developed. However, in some cases, the pericardial effusion, although significant, is not accompanied by other clinically relevant signs of HT. In these children, the pericardial effusion is not recognized or is identified accidentally during radiological tests performed for different clinical reasons. Most cases of pericardial effusion accompanying HT are diagnosed in children with syndromes, such as Down syndrome, that are frequently associated with heart disease and/or with thyroid malfunction and that routinely undergo laboratory and radiological tests to exclude these problems [8,9,10]. However, when it is not promptly identified and treated, the fluid accumulation can lead to unexpected heart problems, including tamponade [8,9,10]. In the case herein reported no other potential cause of pericarditis such as congenital or acquired heart disease of myocardial infection was reported. This indicated that hypothyroidism was the only cause of fluid accumulation in the pericardial space and confirms the diagnostic difficulties that can be found in some cases of pediatric autoimmune hypothyroidism. The fluid accumulation was accidentally identified. Fortunately, although quantitatively relevant, in our patient, it remained asymptomatic and responded promptly to hormonal treatment. However, this case shows that when fluid is identified in the pericardial space and pericarditis of unknown origin is diagnosed, the thyroid function should be immediately evaluated to prescribe substitutive hormonal therapy if necessary and thereby avoid overt hypothyroidism development and the risk of cardiac tamponade. In this child, the need for immediate thyroid function tests was suggested by the presence of a palpable thyroid, which remained undetected despite several visits for recurrent abdominal pain and despite the family history. As previously reported, two close relatives of the child were reported to suffer from HT. Although the aetiology and pathogenesis of HT are not precisely defined, a strong genetic susceptibility to the disease has been confirmed by several family and twin studies. HT occurrence, progression, and severity have been found associated with several genes, including genes for human leukocyte antigen, cytotoxic T lymphocyte antigen-4, protein tyrosine phosphatase nonreceptor-type 22, the vitamin D receptor, thyroglobulin, and cytokines [14]. Environmental factors such as infections, drugs (amiodarone, lithium, interferon-alpha), hormones (oestrogen), dietary components (iodine, selenium), environmental toxins, stress and smoking are considered potential triggers of HT, possibly influencing gene expression [5].

The relevant amount of fluid evidenced in the pericardial space suggests that hypothyroidism had long been established. On the other hand, the comparison of body growth parameters before and after therapy clearly indicates that restoring normal hormonal levels through LT4 administration significantly reduced the body weight and BMI and increased the height velocity to even higher than the maximum reported for normal children of the same age. Hypothyroidism is associated with relevant modifications of growth with a reduced height and an increased weight [1]. In the child herein reported, these growth parameters at admission were both in the normal range, although the height was near the lowest normal values and the body weight and BMI near the highest. This may explain why, despite the family history, no attention was paid to the dissociation of the growth parameters and why hypothyroidism was suspected and finally diagnosed only after discovery of the pericardial effusion. On the other hand, autoimmune hypothyroidism is very rare in children younger than five years of age.

## 5. Conclusions

In conclusion, pericardial effusion can be a sign of several diseases. Paediatricians should not forget that, in some cases, it can be a manifestation of HT, even when other signs of hypothyroidism are absent or poorly evident. Immediate thyroid function tests and adequate hormonal replacement can prevent more-severe heart problems and overt hypothyroidism development.
